# Hepatocyte-derived exosomal miR-146a-5p inhibits hepatic stellate cell EMT process: a crosstalk between hepatocytes and hepatic stellate cells

**DOI:** 10.1038/s41420-023-01602-y

**Published:** 2023-08-19

**Authors:** Zhichao Lang, Yifei Li, Lifan Lin, Xinmiao Li, Qiqi Tao, Yuhang Hu, Menglu Bao, Lei Zheng, Zhengping Yu, Jianjian Zheng

**Affiliations:** 1 Key Laboratory of Clinical Laboratory Diagnosis and Translational Research of Zhejiang Province, The First Affiliated Hospital of Wenzhou Medical University, Wenzhou, 325000 China; 2 Cixi Biomedical Research Institute, Wenzhou Medical University, Ningbo, 315000 China; 3 Laboratory Medicine Center, Nanfang Hospital, Southern Medical University, Guangzhou, 510515 China; 4 Department of Hepatobiliary Surgery, The First Affiliated Hospital of Wenzhou Medical University, Wenzhou, 325000 China

**Keywords:** miRNAs, Protein-protein interaction networks

## Abstract

Recently, Salidroside (Sal) has been demonstrated to suppress hepatic stellate cell (HSC) activation, a crucial event for liver fibrosis. Moreover, Sal has been reported to decrease hepatocyte injury. A growing number of reports have indicated that the crosstalk between hepatocytes and HSCs is very crucial for liver fibrosis development. Whether Sal-treated hepatocytes could inhibit HSC activation is unclear. Exosomes, as vital vehicles of intercellular communication, have been shown to transfer cargos between hepatocytes and HSCs. Herein, we aimed to investigate the roles of exosomal miRNAs from Sal-treated hepatocytes in HSC activation as well as liver fibrosis. Our results showed that Sal suppressed carbon tetrachloride (CCl_4_)-induced liver fibrosis in vivo. HSC activation as well as cell proliferation was repressed in HSCs co-cultured with Sal-treated hepatocytes. Interestingly, miR-146a-5p was up-regulated by Sal in CCl_4_-treated mice. Also, enhanced miR-146a-5p was found in hepatocytes isolated from Sal-treated CCl_4_ mice and hepatocyte-derived exosomes. Notably, hepatocyte exosomal miR-146a-5p contributed to HSC inactivation. Inhibiting miR-146a-5p in hepatocyte exosomes resulted in reduced E-cadherin (E-cad) and increased desmin in HSCs, indicating that miR-146a-5p caused HSC inactivation via epithelial-mesenchymal transition (EMT). miR-146a-5p inhibition-mediated HSC activation and EMT process were blocked down by loss of EIF5A2. Further studies revealed that EIF5A2 was a target of miR-146a-5p. Furthermore, exosomes with miR-146a-5p overexpression inhibited liver fibrosis in CCl_4_ mice. Collectively, exosomal miR-146a-5p from Sal-treated hepatocytes inhibits HSC activation and liver fibrosis, at least in part, by suppressing EIF5A2 and EMT process.

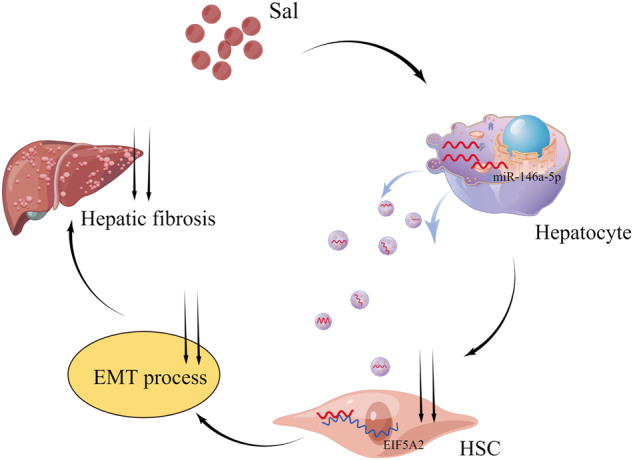

## Introduction

Liver fibrosis, a wound-healing process, is generally caused by chronic liver damages such as viral hepatitis as well as metabolic diseases. Continuous liver damage contributes to fibrosis and even hepatocellular carcinoma [1, 2]. One of the crucial events of liver fibrosis, characterized by excessive extracellular matrix proteins, is hepatic stellate cells (HSCs) activation [3, 4]. Epithelial-mesenchymal transition (EMT), a reversible procedure, is the transformation of epithelial cells into a mesenchymal state [5]. EMT process has been reported to play a crucial role in fibrosis as well as cancer development [6–8]. Recent studies have demonstrated the involvement of EMT process in HSC activation [9].

It is known that traditional Chinese medicine contributes to the inhibition of liver fibrosis development including liver inflammation, hepatic blood flow and liver regeneration [10]. Salidroside (Sal), a phenolic compound and also main active component in plants of Rhodiola rosea L, exhibits a series of pharmacological properties, such as anti-inflammation, anti-oxidation and anti-cancer [11–13]. Recently, Sal has been shown to have curative effects on liver injury, hepatocyte apoptosis, and liver fibrosis. For instance, Sal has been demonstrated to repress insulin resistance and hepatic steatosis via down-regulating miR-21 in high fat diet-fed rats [14]. Additionally, Sal has been shown to suppress HSC activation as well as autophagy [15].

Exosomes, 40-100 nm in size, are small membrane vesicles that have the same topology as the cells [16]. Generally, exosomes, released by various cells into biological fluids, carry contents like microRNAs (miRNAs), proteins and mRNAs. With a large number of bioactive molecules, exosomes mediate information exchange among cells. Increasing evidence has shown the vital roles of exosomes in many cellular processes [17]. For example, Zhang et al. previously reported that exosomes arising from hepatitis B virus contribute to liver fibrosis progression through miR-222/TFRC axis [18]. Therefore, exosomes participate in liver fibrosis development.

miRNAs, the members of the family of post-transcriptional gene repressors, have been widely related to the adjustment of gene expression in diverse surroundings, encompassing almost all respects of metabolism about systemic control [19]. Recent studies have shown that miRNAs could modulate physiological and pathological functions of the liver. Dysregulation of miRNAs is closely associated with liver fibrosis, liver metabolism imbalance, liver injury and tumor development [20]. Currently, miRNAs have been found to act as crucial therapeutic factors for the diagnosis and treatment of hepatic fibrosis. Du et al. previously reported that miR-146a-5p inhibits activation of HSCs via suppressing Wnt1 and Wnt5a [21]. Chiabotto et al. identified miR-146a-5p as a key component of human liver stem cell (HLSC) exosomes that have an inhibitory effect on HSC activation [22]. Recently, Sal has been shown to promote the secretion of miR-146a-5p via exosomes by epithelial-like cells that have an effect on macrophage-like cells [23]. Combined with these, it is interesting whether exosomal miR-146a-5p derived from Sal-treated hepatocyte has inhibitory effects on the activation of HSCs.

## Results

### Sal alleviates CCl_4_-induced liver fibrosis in mice

**Fig. 1:**
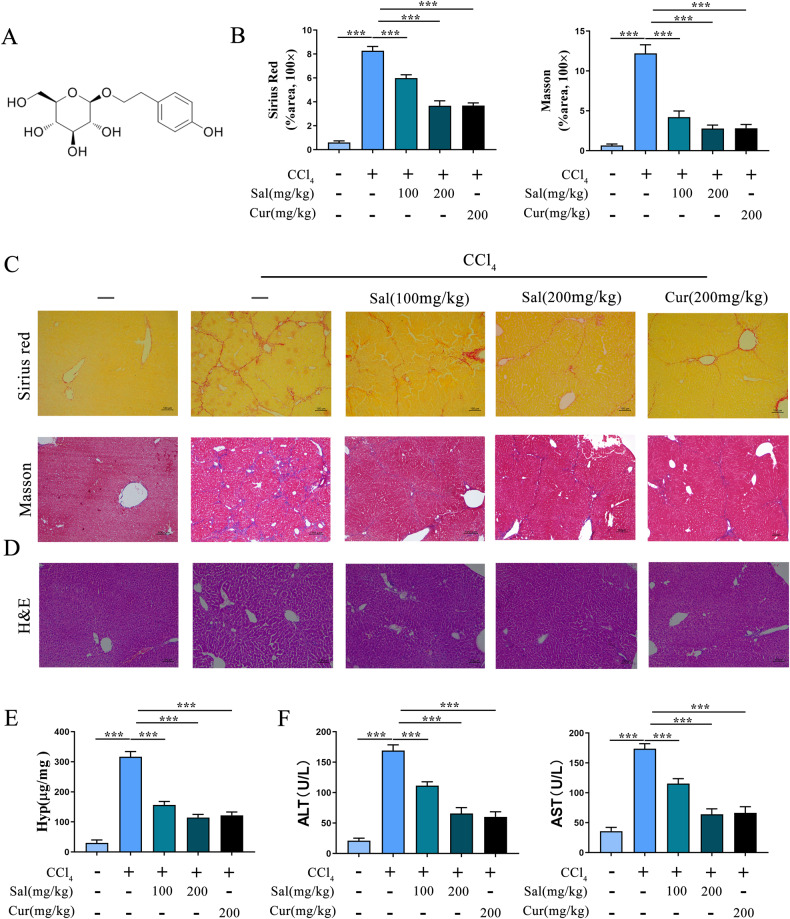
Sal alleviates CCl_4_-induced liver fibrosis in mice. CCl_4_-induced mice were treated with Sal (100 mg/kg or 200 mg/kg) or Cur (200 mg/kg). **A** Chemical structure of Sal. **B** The quantitative results of Sirius red staining and Masson staining. **C** Sirius red staining and Masson staining were used to evaluate collagen deposition. Scale bar, 100 μm. **D** HE staining. Scale bar, 100 μm. **E**, **F** Levels of Hyp, ALT and AST. Each value is the mean ± SD of six experiments. ***p < 0.001.

A classical mouse model of liver fibrosis induced by CCl_4_ was established to explore the effects of Sal on liver fibrosis. Compared with the control, CCl_4_ induced typical hepatic fibrosis characteristics in mice, with an increase in collagen production (Fig. 1B, C). HE staining revealed that CCl_4_ destroyed liver normal structure (Fig. 1D). As shown in Fig. 1E, results of Hyp revealed that CCl_4_ caused a significant increase in Hyp level, suggesting the enhancement of collagen by CCl_4_. In line with it, serum ALT and AST were enhanced by CCl_4_ (Fig. 1F). All the data suggest the establishment of CCl_4_ model. However, the effects of CCl_4_ on liver fibrosis were inhibited by Sal (Fig. 1B–F). To confirm the inhibitory effects of Sal, Cur (an effective anti-fibrosis drug) was used as a positive control. Cur additionally inhibited CCl_4_-induced liver fibrosis in mice (Fig. 1B–F). Our results suggest that Sal contributes to suppressing liver fibrosis in vivo.

### Sal-treated hepatocytes suppress HSC activation

**Fig. 2:**
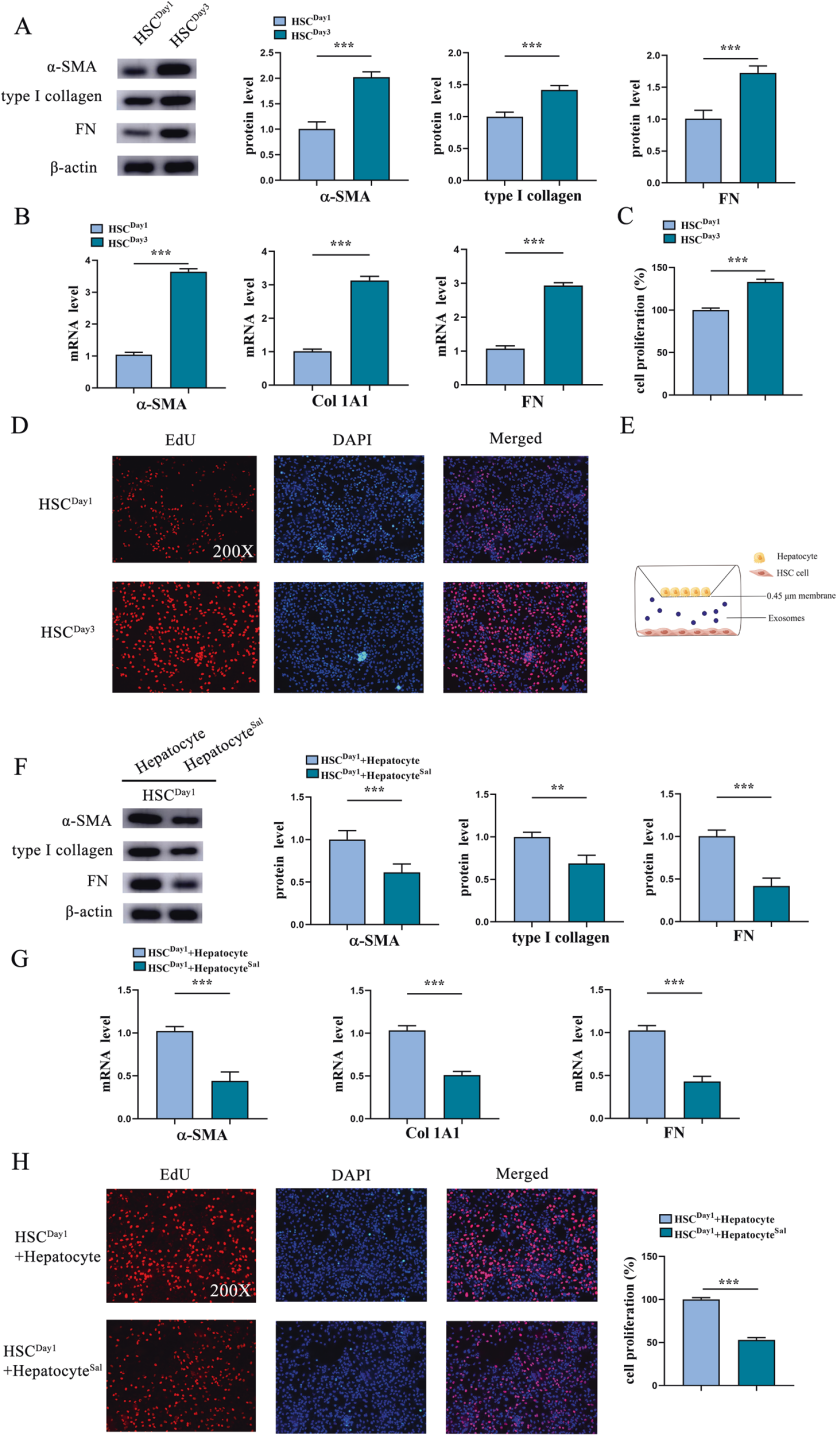
Sal-treated hepatocytes suppress HSC activation. Primary HSCs were isolated from healthy mice. **A** Protein expressions and (**B**) mRNA expressions of α-SMA, Col1A1 and FN in HSCs at day 1 and day 3. **C** The quantitative results of EdU assay of cell proliferation in HSCs at day 1 and day 3. **D** EdU assay of cell proliferation in HSCs at day 1 and day 3. **E** Illustration of the indirect co-culture system: HSCs co-cultured with Sal-treated hepatocytes, using Figdraw. **F** Protein expressions and (**G**) mRNA expressions of α-SMA, Col1A1 and FN in HSCs co-cultured with Sal-treated hepatocytes. **H** EdU assay of cell proliferation in HSCs co-cultured with Sal-treated hepatocytes. Each value is the mean ± SD of three experiments. **p < 0.01, ***p < 0.001.

Due to the importance crosstalk between hepatocytes and HSCs as well as the inhibitory effect of Sal on liver fibrosis development [15, 24], it is interesting whether Sal-treated hepatocytes have inhibitory effects on HSC activation. Firstly, we isolated primary HSCs from healthy mice. Generally, isolated primary HSCs will be gradually activated during culture time. Herein, we found that compared with primary 1-day-old HSCs, enhanced fibrotic markers including α-SMA, collagen type I alpha 1 chain (Col1A1) and FN were found in HSCs at Day 3 (Fig. 2A, B). In line with it, results of EdU assays showed an increase in cell proliferation in primary 3-day-old HSCs compared with primary 1-day-old HSCs (Fig. 2C, D). Next, to determine the effects of Sal-treated hepatocytes on HSC activation, fibrosis-related genes such as α-SMA, Col1A1 and FN were examined in HSCs co-cultured with Sal-treated hepatocytes (Fig. 2E). The co-culture between hepatocytes and HSCs resulted in HSC inactivation, including reduced fibrosis-related genes and proliferation rate (Fig. 2F–H). Combined with these, we demonstrate that Sal-treated hepatocytes contribute to HSC inactivation.

### miR-146a-5p is up-regulated by Sal

**Fig. 3:**
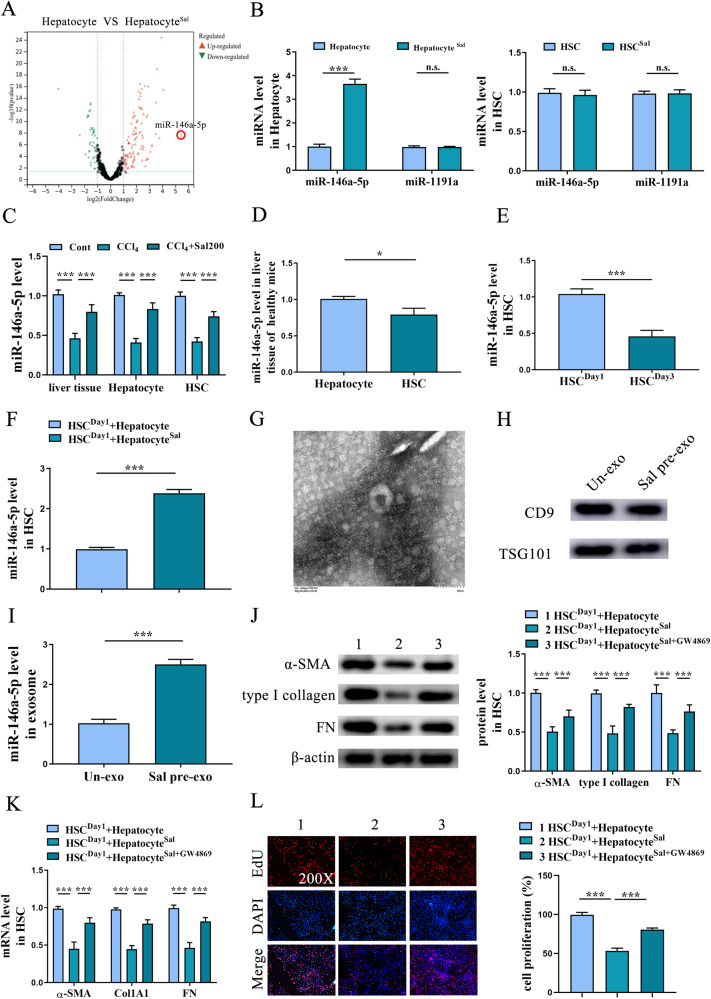
Up-regulation of miR-146a-5p in exosomes of Sal-treated hepatocytes. **A** Volcano plot displayed changed miRNAs in hepatocytes after Sal (100 µM) treatment group. **B** Expression of miR-146a-5p and miR-1191a in hepatocytes and HSCs after Sal treatment. **C** Expression of miR-146a-5p in liver tissue, isolated hepatocytes and HSCs from CCl_4_ mice with Sal treatment (200 mg/kg). **D** Expression of miR-146a-5p in hepatocytes and HSCs isolated from healthy mice. **E** Expression of miR-146a-5p in HSCs at day 1 and day 3. **F** Expression of miR-146a-5p in HSCs co-cultured with Sal-treated hepatocytes. **G** Isolated exosomes from hepatocytes visualized by TEM. **H** The protein expressions of CD9 and TSG101 in exosomes. **I** Expression of miR-146a-5p in Un-exo and Sal pre-exo groups. **J** Protein expressions and (**K**) mRNA expressions of α-SMA, Col1A1 and FN in HSCs co-cultured with GW4869 (10 µM)-treated hepatocytes after Sal treatment. **L** EdU assay of cell proliferation in HSCs co-cultured with GW4869-treated hepatocytes after Sal treatment. Cont, the control group; CCl_4_, the CCl_4_ group; CCl_4_+Sal200, mice given 200 mg/kg Sal treatment; Un-exo, exosomes from hepatocytes; Sal pre-exo, exosomes from Sal-treated hepatocytes. Each value is the mean ± SD of three experiments. *p < 0.05, ***p < 0.001, ns, no significant.

The underlying molecular mechanism for Sal-treated hepatocytes-mediated HSC inactivation was subsequently explored. Previously, Zheng et al. found that Sal could suppress the inflammation activity of alveolar macrophages via enhancing the secretion of exosomal miR-146a-5p in pulmonary epithelial cells [23]. Whether miR-146a-5p participates in the effect of Sal-treated hepatocytes on HSC inactivation was investigated. Notably, hepatocyte miRNA-sequence analysis indicated that miR-146a-5p was predominately increased among all the up-regulated miRNAs (Fig. 3A). qRT-PCR analysis confirmed that miR-146a-5p was obviously increased by Sal in hepatocytes, whereas HSCs not (Fig. 3B). However, miR-1191a, as an unrelated control, was no change in cells (Fig. 3B). We additionally found that in vivo, there was a significant increase in miR-146a-5p in the livers from CCl_4_ mice after Sal treatment (Fig. 3C). In line with it, enhanced miR-146a-5p was found in primary hepatocytes and primary HSCs isolated from mice after Sal treatment (Fig. 3C). Consistent with it, higher miR-146a-5p was shown in primary hepatocytes isolated from the healthy mice compared with primary HSCs (Fig. 3D). Moreover, miR-146a-5p was gradually reduced in HSCs during culture time (Fig. 3E). These results collectively suggest that increased miR-146a-5p in HSCs may be from hepatocytes. Further studies confirmed that increased miR-146a-5p was shown in HSCs co-cultured with Sal-treated hepatocytes (Fig. 3F). Our data reveal the induction of miR-146a-5p by Sal in vivo and in vitro.

### Up-regulation of miR-146a-5p in exosomes of Sal-treated hepatocytes

Increasing studies have revealed that miRNAs could be transported by exosomes among different cells. Therefore, we hypothesized that enhanced miR-146a-5p in HSCs is derived from exosomes of Sal-treated hepatocytes. As shown in Fig. 3G, results of TEM confirmed the classic shape of exosomes in the supernatant of Sal-treated hepatocytes. Exosome markers such as CD9 and TSG101 were also detected by western blot (Fig. 3H), suggesting successful extraction of exosomes. As expected, up-regulation of miR-146a-5p was shown in exosomes from Sal-treated hepatocytes (Sal pre-exo) in comparison with the control exosomes from hepatocytes (Un-exo) (Fig. 3I). GW4869, a widely used pharmacological agent, has been reported to inhibit exosome generation. To further determine the role of exosomes from Sal-treated hepatocytes in HSC activation, hepatocytes were treated with GW4869. Interestingly, the expressions of α-SMA, Col1A1 and FN were enhanced in HSCs co-cultured with GW4869-treated hepatocytes after Sal treatment in comparison with HSC co-cultured with Sal-treated hepatocytes (Fig. 3J and Fig. 3K). In line with it, results of EdU assays showed an increase in HSC proliferation after GW4869 treatment (Fig. 3L). These results were confirmed the crucial role of hepatocyte exosome in HSC inactivation. Taken together, increased miR-146a-5p in HSCs may be from exosomal miR-146a-5p of Sal-treated hepatocytes.

### Exosomal miR-146a-5p suppresses HSC activation

**Fig. 4:**
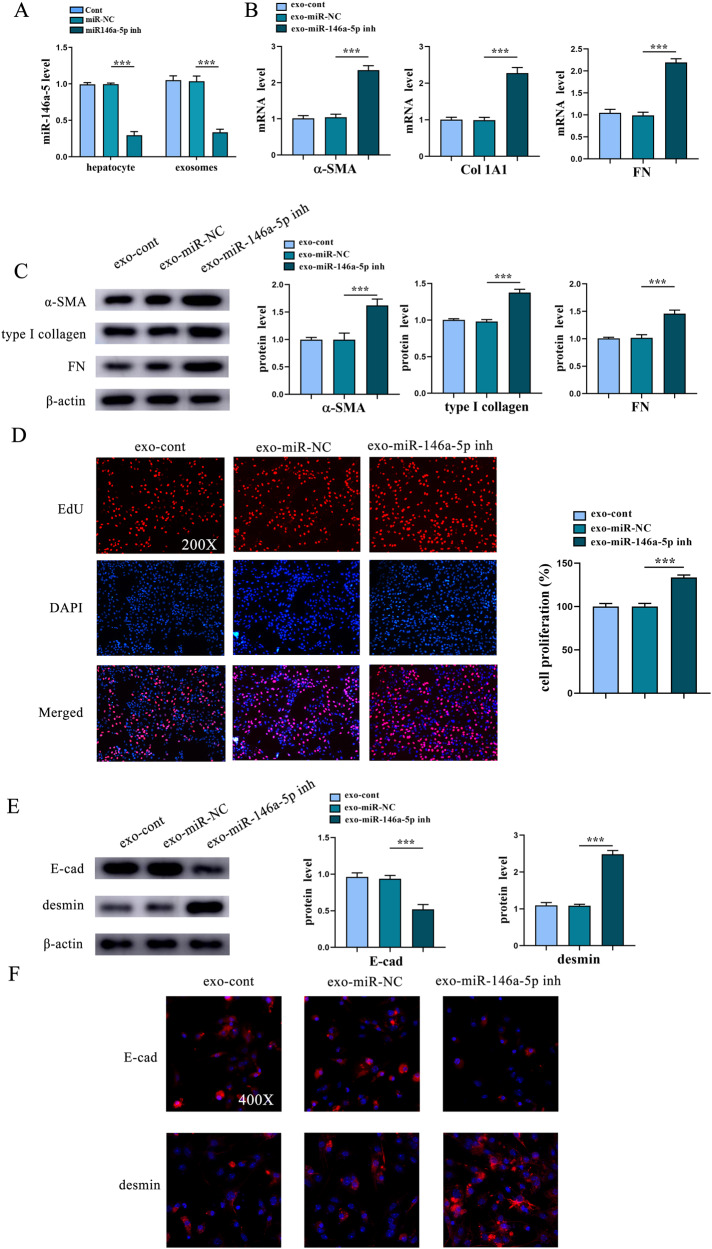
Exosomal miR-146a-5p suppresses HSC activation. Primary 1-day-old HSCs were treated with exosomes from hepatocytes after miR-146a-5p inhibitor transfection. **A** Expression of miR-146a-5p in hepatocytes and exosomes from hepatocytes with exo-miR-146a-5p inh treatment. **B** mRNA expressions and (**C**) protein expressions of α-SMA, Col1A1 and FN in HSCs. **D** EdU assay of cell proliferation in HSCs. **E** Protein expressions of E-cad and desmin in HSCs after exo-miR-146a-5p inh treatment. **F** Immunofluorescence staining for E-cad (red) and desmin (red). DAPI stained the nuclei blue. Exo-cont, HSCs treated with exosomes from hepatocytes; exo-miR-NC, HSCs treated with exosomes from hepatocytes after miR-NC transfection; exo-miR-146a-5p inh, HSCs treated with exosomes from hepatocytes after miR-146a-5p inhibitor transfection. Each value is the mean ± SD of three experiments. ***p < 0.001.

To address whether exosomal miR-146a-5p from hepatocytes acts as a regulator of HSC activation, miR-146a-5p inhibitor was transfected into hepatocytes. Compared with the control, miR-146a-5p was reduced in hepatocytes and exo-miR-146a-5p inh (Fig. 4A). Next, the fibrosis-related genes were examined in HSCs with exo-miR-146a-5p inh. Loss of miR-146a-5p induced the expressions of α-SMA, Col1A1 and FN compared with the untreated HSCs (Fig. 4B, C). Loss of miR-146a-5p additionally promoted HSC proliferation (Fig. 4D). Notably, exo-miR-146a-5p inh promoted EMT process, with reduced E-cad and increased desmin (Fig. 4E). Immunofluorescence analysis further confirmed reduced E-cad (red) as well as enhanced desmin (red) in HSCs with exo-miR-146a-5p inh (Fig. 4F). Our results reveal the importance of exosomal miR-146a-5p in the crosstalk between Sal-treated hepatocytes and HSCs, and the involvement of EMT process in the biological role of miR-146a-5p in HSCs.

### miR-146a-5p inhibits HSC EMT process via targeting EIF5A2

**Fig. 5:**
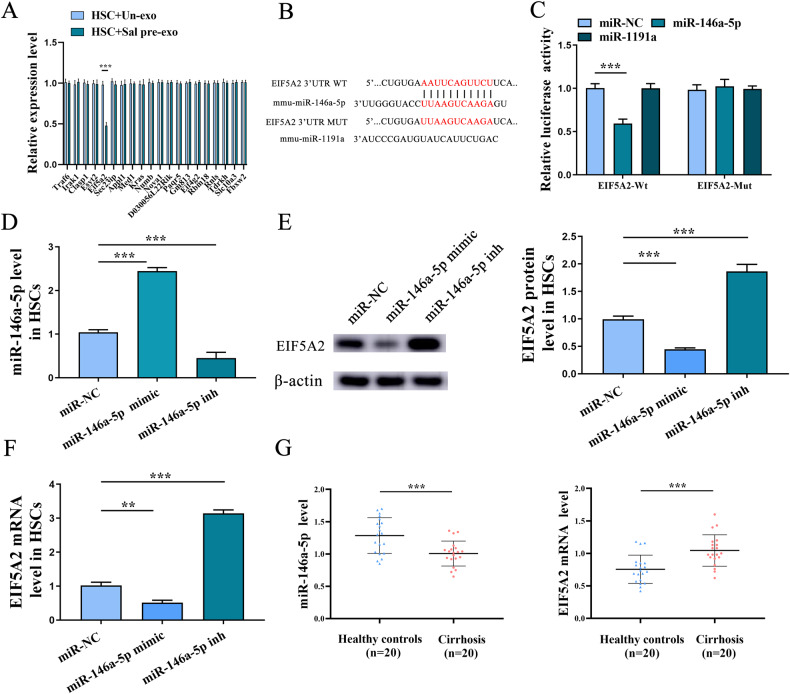
miR-146a-5p inhibits HSC EMT process via targeting EIF5A2. **A** Expressions of predicted genes in HSCs co-cultured with Un-exo or Sal pre-exo. **B** The binding sites of EIF5A2 3′ UTR with miR-146a-5p. **C** Luciferase reporter assays. **D** Expression of miR-146a-5p in HSCs with miR-146a-5p mimic or inhibitor. **E** Protein expression of EIF5A2 in HSCs with miR-146a-5p mimic or inhibitor transfection. **F** The mRNA expression of EIF5A2 in HSCs with miR-146a-5p mimic or inhibitor. **G** Expressions of miR-146a-5p and EIF5A2 in serum samples from healthy controls and patients with cirrhosis. HSC+Un-exo, HSCs co-cultured with exosomes from hepatocytes; HSC+Sal pre-exo, HSCs co-cultured with exosomes from Sal-treated hepatocytes. Each value is the mean ± SD of three experiments. **p < 0.01, ***p < 0.001.

Bioinformatic analysis was performed to determine potential targets of miR-146a-5p. In addition, the underlying mechanism responsible for the inhibitory role of miR-146a-5p in EMT process as well as HSC activation was also explored. To identify the potential targets of miR-146a-5p, bioinformatics analysis (miRDB) was then performed. It was found that there were 20 predicted genes with the highest score. We found that only EIF5A2 was significantly down-regulated in HSCs co-cultured with exosomes of Sal-treated hepatocytes, whereas other predicted genes not (Fig. 5A). Therefore, EIF5A2 was selected for the next studies. Using pmirGLO, we generated a EIF5A2 luciferase reporter, EIF5A2-Wt or EIF5A2-Mut, containing the miR-146a-5p-binding sites (Fig. 5B). Analysis of luciferase activities showed that miR-146a-5p resulted in a reduction in the luciferase activity of EIF5A2-Wt and had no effect on EIF5A2-Mut, indicating that EIF5A2 is a target of miR-146a-5p (Fig. 5C). Moreover, miR-1191a had no effect on the luciferase activity of both EIF5A2-Wt and EIF5A2-Mut (Fig. 5C). Next, whether miR-146a-5p targets EIF5A2 was further studied. It was found that miR-146a-5p was up-regulated and down-regulated in HSCs transfected with miR-146a-5p mimic and inhibitor, respectively (Fig. 5D). Then, miR-146a-5p mimic led to a reduction in EIF5A2 in HSCs, while miR-146a-5p inhibitor contributed to the enhancement of EIF5A2 (Fig. 5E, F). Accordingly, we found that decreased miR-146a-5p as well as increased EIF5A2 was found in patients with cirrhosis in comparison with the healthy controls (Fig. 5G).

**Fig. 6:**
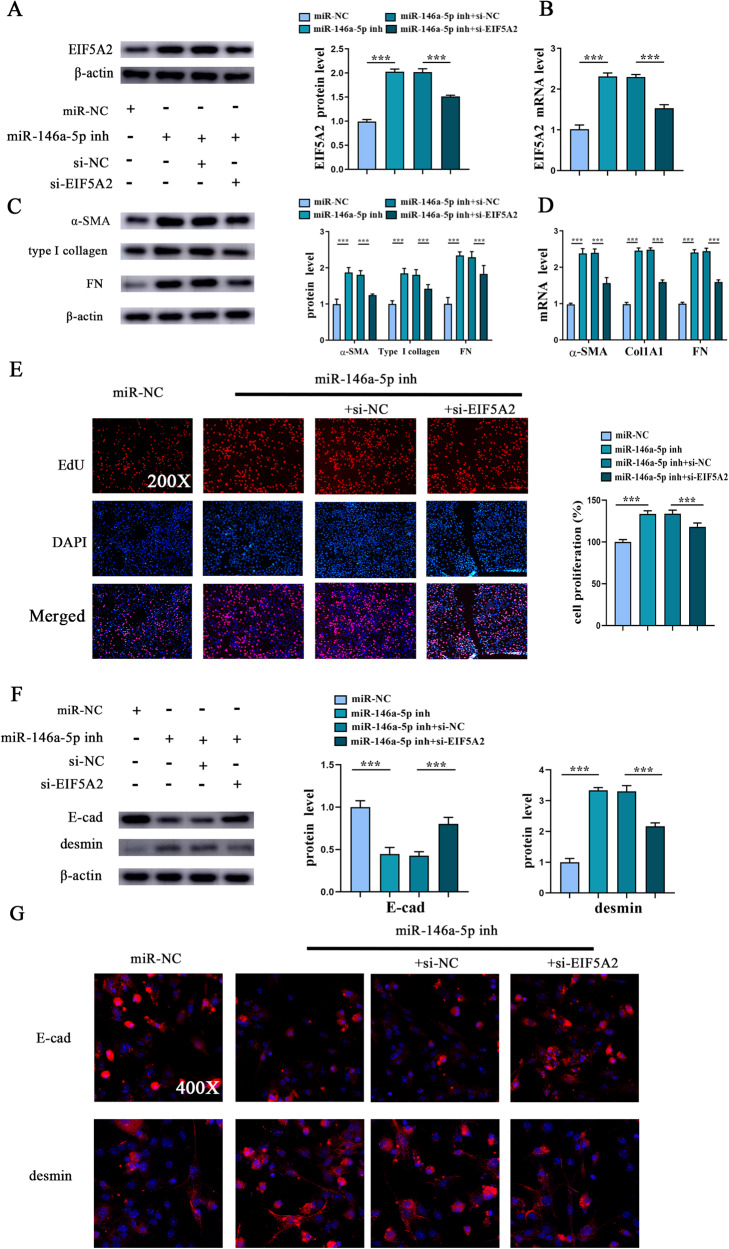
miR-146a-5p inhibits EMT process during HSC activation via targeting EIF5A2. Primary 1-day-old HSCs were treated with miR-146a-5p inhibitor and additionally transfected with si-EIF5A2. **A** Protein expression and (**B**) mRNA expression of EIF5A2 in miR-146a-5p inhibitor-treated HSCs after si-EIF5A2 transfection. **C** Protein expressions and (**D**) mRNA expressions of α-SMA, Col1A1 and FN in miR-146a-5p inhibitor-treated HSCs after si-EIF5A2 transfection. **E** EdU assay of cell proliferation in miR-146a-5p inhibitor-treated HSCs after si-EIF5A2 transfection. **F** Protein expression of E-cad and desmin in miR-146a-5p inhibitor-treated HSCs after si-EIF5A2 transfection. **G** Immunofluorescence staining for E-cad (red) and desmin (red) in miR-146a-5p inhibitor-treated HSCs after si-EIF5A2 transfection. DAPI stained the nuclei blue. Each value is the mean ± SD of three experiments. ***p < 0.001.

Next, whether EIF5A2 is involved in miR-146a-5p-inhibited HSC activation and EMT process was explored. si-EIF5A2 was transfected into HSCs with miR-146a-5p inhibitor. Clearly, in HSCs with miR-146a-5p inhibitor, miR-146a-5p inhibition-induced EIF5A2 was inhibited by si-EIF5A2 (Fig. 6A, B). Then, miR-146a-5p inhibition-caused HSC activation including enhanced α-SMA, Col1A1 and FN, was blocked down by loss of EIF5A2 (Fig. 6C, D). In line with it, miR-146a-5p inhibition-induced HSC proliferation was also suppressed by silencing EIF5A2 (Fig. 6E). We additionally explored whether EIF5A2 is responsible for miR-146a-5p-mediated EMT process. Clearly, results of western blot showed that reduced E-cad as well as increased desmin caused by miR-146a-5p inhibition was suppressed by loss of EIF5A2 (Fig. 6F). Likewise, similar results were found in analysis of immunofluorescence (Fig. 6G). All the data suggest that miR-146a-5p inhibits EMT process during HSC activation.

### Exosomal miR-146a-5p mitigates liver fibrosis in vivo

**Fig. 7:**
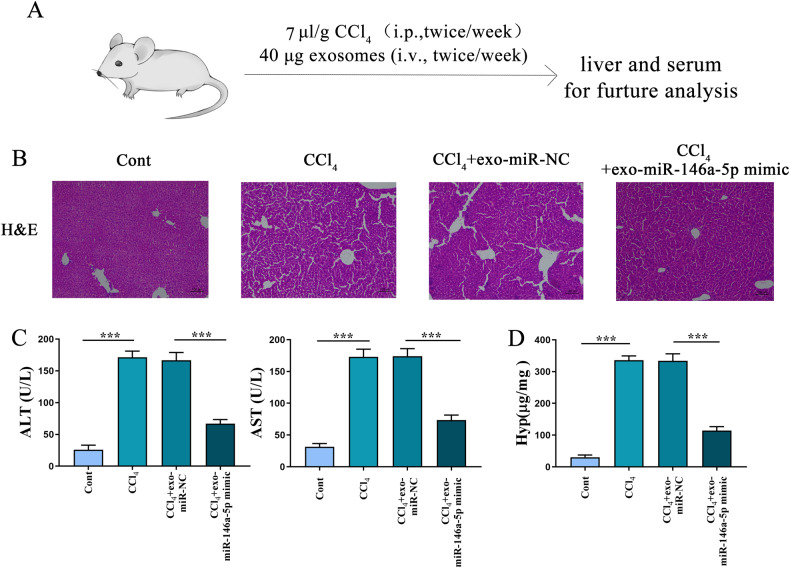
Exosomal miR-146a-5p mitigates liver fibrosis in vivo. CCl_4_-induced mice were treated with exosomes (40 μg) from hepatocytes with miR-146a-5p mimic transfection. **A** The exosomes treatment strategy for CCl_4_-induced liver fibrosis in vivo. **B** HE staining. Scale bar, 100 μm. **C**, **D** Levels of ALT, AST and Hyp. Cont, the control group; CCl_4_, the CCl_4_ group; CCl_4_+exo-miR-NC, CCl_4_ mice after exosomes from hepatocytes with miR-NC transfection; CCl_4_+exo-miR-146a-5p mimic, CCl_4_ mice after exosomes from hepatocytes with miR-146a-5p mimic transfection. Each value is the mean ± SD of six experiments. ***p < 0.001.

Finally, we assessed the effect of exosomal miR-146a-5p on CCl_4_-induced liver fibrosis in vivo. CCl_4_ mice were treated with the exo-miR-146a-5p mimic (Fig. 7A). Results of HE staining showed that exo-miR-146a-5p mimic mitigated liver fibrosis in CCl_4_ mice (Fig. 7B). In addition, compared with the control, the level of liver injury caused by CCl_4_ was suppressed by exo-miR-146a-5p mimic (Fig. 7C). Results of Hyp content revealed reduced collagen deposition in CCl_4_ mice with exo-miR-146a-5p mimic (Fig. 7D). In sum, our data suggest the inhibitory role of exosomal miR-146a-5p in liver fibrosis.

## Discussion

Hepatocytes, the most abundant parenchymal cells and the main responsors upon inflammatory stimulus, consist of 90% of liver biomass. It is known that chemokines, produced by hepatocytes, recruit immune cells to participate in disease progression [25]. HSCs, resident non-parenchymal liver pericytes, have been demonstrated to act as a key liver fibrosis-related factor. Upon liver injury, HSCs will be activated or transdifferentiated from a static state into proliferative, motional myofibroblasts that secrete extracellular matrix [26]. Increasing evidence has shown the importance of the crosstalk between hepatocytes and HSCs during liver fibrosis. However, the underlying mechanism of the crosstalk between hepatocytes and HSCs remains largely unclear. In this study, owing to elevated miR-146a-5p in exosomes from Sal-treated hepatocytes, miR-146a-5p was increased in HSCs and suppressed EIF5A2 expression, leading to the suppression of EMT process as well as HSC inactivation. Hepatocyte exosomal miR-146a-5p contributes to the suppression of HSC activation via regulation of EIF5A2 and EMT process, and this is a first report.

Sal, a natural compound obtained from Chinese herbs of the genus Rhodiola, has demonstrated to inhibit apoptosis and autophagy [27]. Previously, Ouyang et al. reported that Sal and mesenchymal stem cell could synergistically improve liver fibrosis [28]. Consistent with the previous study, Sal was shown to have an inhibitory effect on CCl_4_-induce liver fibrosis, with a reduction in collagen level and ALT/AST. Chiabotto et al. revealed the inhibitory role of HLSC exosomal miR-146a-5p in HSCs [22]. Herein, our results firstly revealed that exosomes are the key crosstalk medium between Sal-treated hepatocytes and HSCs. Activation of HSC is inhibited by Sal, at least in part, via hepatocyte exosomal miR-146a-5p, which is a novel mechanism suppressing liver fibrosis.

Exosomes play a key role in intercellular crosstalk by delivering the instructional payload, such as mRNAs, miRNAs and proteins [29, 30]. miRNAs have been shown to act as key factors in the transformation of HSC fibrotic phenotypes [31]. For instance, miR-146a-5p has been reported to act as an anti-fibrotic factor in irradiated and TGF-β1-stimulated LX2 cells via PTPRA-SRC signaling [32]. In this study, miR-146a-5p, reduced in activated HSCs, had an inhibitory effect on HSC activation, which is consistent with the previous study [21]. Subsequently, increased miR-146a-5p in HSCs was confirmed to be associated with exosomes from Sal-treated hepatocytes, which was shown to inhibit HSC activation. Further studies showed that exosomal miR-146a-5p from Sal-treated hepatocytes effectively reduced HSC proliferation, activation and EMT process via regulation of EIF5A2.

EIF5A, a highly conserved protein, has been shown to be involved in mRNA translation, cellular proliferation and inflammation [33]. In addition, EIF5A2 is crucial for maintaining polyamine homeostasis [33, 34]. Recently, EIF5A2 contributes to enhancing the EMT process of tumors, resulting in the progression and metastasis of cancers [35, 36]. Zhu et al. previously demonstrated that EIF5A2 induces colorectal carcinoma cell EMT, leading to enhanced invasiveness of cancer cells [37]. EMT/MET are known to occur when tissues are constructed during embryogenesis/development. They are also thought to occur during adult tissue remodeling responses, including carcinogenesis and fibrosis. During culture, several resident adult liver cells appear capable of undergoing EMT and/or MET, raising the possibility that EMT/MET might be involved in liver regeneration. When EMT activity outstrips MET, repair is mainly fibrogenic, causing liver fibrosis. Conversely, predominance of MET favors more normal liver regeneration [38]. Herein, bioinformatics analysis predicted that EIF5A2 may be a target of miR-146a-5p, which was confirmed by further studies. Notably, miR-146a-5p inhibition-mediated HSC activation and EMT process were blocked down by loss of EIF5A2. Taken together, the present study revealed a novel mechanism of miR-146a-5p-inhibited HSC activation. Additionally, a negative correlation between EIF5A2 and miR-146a-5p was found in HSCs and patients with cirrhosis. Collectively, we demonstrate that exosomal miR-146a-5p inhibits HSC EMT process, at least in part, via its target EIF5A2.

## Conclusion

In conclusion, exosomal miR-146a-5p from Sal-treated hepatocytes inhibits HSC activation and liver fibrosis, at least in part, via suppressing EIF5A2 and EMT process.

## Materials and methods

### Clinical samples collection

Serum samples of 20 patients with liver cirrhosis and 20 normal participants (with normal liver biochemistry, no history of liver disease or alcohol abuse and no viral hepatitis) were collected from the First Affiliated Hospital of Wenzhou Medical University (FAHWMU). The FAHWMU Ethics Committee reviewed and approved this study. Informed consents were obtained from all participants before obtaining the samples.

### Animal treatments

C57BL/6 J mice were administered carbon tetrachloride (CCl_4_) (Sigma) using olive oil (10%, 7 µL/g mice) twice per week for a total of 8 weeks (*n* = 6) [39]. The allocation of mice in each group were randomized and blinded. Control group (*n* = 6) received the identical volume of olive oil. Sal was purchased from Sigma (Fig.1A). The Sal group consisted of mice (*n* = 6) given Sal (100 mg/kg) and mice (*n* = 6) given Sal (200 mg/kg) daily via gavage during CCl_4_ period. Additionally, Curcumin (Cur) group consisted of mice (*n* = 6) given Cur (200 mg/kg) daily via gavage during CCl_4_ period.

For tail vein injection, forty micrograms exosomes with miR-146a-5p overexpression (*n* = 6) or miRNA negative control (miR-NC) (*n* = 6) were administered two injection per week for 8 weeks during CCl_4_ period [40]. The exosomes were isolated from the supernatant of hepatocyte culture medium with miR-146a-5p mimic transfection.

### Cell culture

Isolation of primary HSCs was conducted from C57BL/6 J mice [41]. Primary hepatocytes isolation was done from mouse livers using a collagenase perfusion method [42]. For co-culture, primary 1-day-old HSCs were seeded into the lower compartment of the Transwell chamber and hepatocytes were inoculated into the upper chamber at a density of 1 × 10^5^ per well. In addition, hepatocytes or primary 1-day-old HSCs were pretreated with Sal (100 µM). Furthermore, primary 1-day-old HSCs, treated with exosomes from hepatocytes with miR-146a-5p mimic (exo-miR-146a-5p mimic) or inhibitor (exo-miR-146a-5p inh) transfection, were washed for further studies. Hepatocytes were treated with Sal (100 µM) and GW4869 (10 µM).

### Histologic analysis

Liver tissues of mice were fixed and then paraffin-embedded, which were further used for Sirius red staining, Masson staining and Hematoxylin and eosin (HE) staining. Leica DM4B microscope was used to capture images.

### Hepatic hydroxyproline (Hyp) content

As described previously [43], based on manufacturer’s instructions, Hydroxyproline Colorimetric Assay kit (Jiancheng Biological Engineering Research Institute) was used to detect the Hyp content in liver tissues.

### Alanine Aminotransferase (ALT) and Aspartate Aminotransferase (AST) assay

From the mice, collection of serum samples was done, followed by the measurement of serum ALT and AST via a commercial kit (Rongsheng).

### Western blot analysis

With the use of RIPA buffer, proteins were extracted. The protein samples were electrophoretically segregated using a 10% SDS-PAGE. Then, the protein samples were transferred onto PVDF membranes and the incubation was performed overnight at 4 °C. The antibodies for Western blotting were: anti-eukaryotic initiation factor 5A2 (EIF5A2) (Abcam), anti-α-smooth muscle actin (α-SMA) (Abcam), anti-fibronectin (FN) (Abcam), anti-type I collagen (Abcam), anti-β-actin (Abcam), anti-CD9 (Abcam), anti-Tumor Susceptibility Gene 101 (TSG101) (Abcam), anti-E-cadherin (E-cad) and anti-desmin antibodies, at a dilution of 1:1000. Next, the second antibody (HRP-conjugated Affinipure goat anti-rabbit IgG; 1:5000 dilution; proteintech) was treated for 1 h at room temperature. Bands were visualized using an ECL chemiluminescent agent (Beyotime). An automatic chemical luminous imaging analysis system was used for capturing images. Quantification of protein expression was via ImageJ software (NIH ImageJ 1.47). β-actin was used as an internal reference to normalize protein expression.

### Quantitative Real-Time PCR (qRT-PCR) analysis

TRIzol reagents (Thermo Fisher) were used to isolate total RNA from cultured cells or liver tissues. According to the manufacturer’s instruction, RNA was transcribed to cDNA. SYBR Green Master Mix (Promega) was used for examining gene expression. The detection of miRNAs was conducted by miRNA Detection kit (GenePharma). RT-PCR was performed using a 7500 Fast system (Applied Biosystems). β-actin and U6 were used as the control to evaluate the relative quantitative of mRNAs and miRNA, respectively. The expression levels (2^−∆∆Ct^) of genes were calculated as described previously [44]. The primers were shown in Table S1.

### 5-Ethyny-2'-Deoxyuridine (EdU) assay

Cell proliferation was detected using EdU assay (RiboBio). HSCs were labeled with EdU for 2 h. A fluorescent microscope (Leica) was used to visualize the EdU^+^ cells.

### miRNA-sequencing (miRNA-seq)

Total RNA was isolated from the mesangial cells using TRIzol® (Invitrogen) per the manufacturer’s instructions. miRNA-Seq was performed using the Illumina HiSeqTM 2000 sequencing system. Expressions of transcripts with |Log_2_ Fold Change| >2, adjusted *P* < 0.05 were deemed statistically significant.

### Exosome extraction and identification

According to the 2018 MISEV guideline [45], exosomes were isolated from hepatocyte supernatant. Transmission electron microscope (TEM) was used to verify exosome themorphology. Then, exosomal marker proteins were examined using western blot analysis, including CD9 and TSG101.

### Cell transfection

Transfection was performed when cell confluence reached 80%. 50 nM miR-146a-5p mimic, miR-146a-5p inhibitor, miR-NC or EIF5A2 short interfering RNA (si-EIF5A2) (Genomeditech) were transfected into hepatocytes or HSCs with serum-free media (non-use of antibiotics) using Lipofectamine 2000 (Invitrogen), respectively. After 6 h, cells were cultured for additionally 48 h in normal media (10% fetal bovine serum and 1% antibiotics) at 37 °C and 5% CO_2_.

### Immunofluorescence analysis

Primary HSCs from mice were immobilized in 4% paraformaldehyde. After fixation, cells were washed with PBS and blocked with 5% BSA. Then, cells were incubated with anti-E-cad (1:100 dilution; Abcam) and anti-desmin (1:100 dilution; Abcam) antibodies overnight at 4 °C. Then, cells were stained with fluorescence-labeled anti-rabbit Alexa 594 (1:50 dilution; Dianova)-conjugated antibodies. For nuclear counterstaining, 4,6-diamidino-2-phenylindole (DAPI) was applied.

### Bioinformatic analysis

The possible targets of miR-146a-5p as well as association between miR-146a-5p and EIF5A2 were predicted using miRDB (https://mirdb.org/).

### Luciferase activity assay

As described previously [43], in order to generate luciferase reporter constructs, the 3′UTR fragment of EIF5A2 wild type (EIF5A2-WT), which encompasses potential sites binding with miR-146a-5p, was cloned into the pmirGLO plasmids (Promega). The EIF5A2 mutant type (EIF5A2-MUT) was also generated. To detect luciferase activity, HEK293 cells were transfected with either WT or MUT plasmid along with miR-146a-5p mimic or miR-1191a mimic using lipofectamine 3000 transfection (Invitrogen). After 48 h, Dual-Luciferase Reporter Assay System (Promega) was used to determine the relative luciferase activity.

### Statistical analysis

Data were presented as the means ± SD. Student’s *t*-test was used to compare differences between two groups. For multiple groups, One-way analysis of variance (one-way ANOVA) was used to analyze the data. *P* < 0.05 was considered significant. All statistical analyses were performed using the SPSS software (version 16.0; SPSS, Chicago, IL).

### Supplementary information

Supplemental Material

Table S1

## Data Availability

The datasets generated during and/or analysed during the current study are available from the corresponding author on reasonable request.
