# Supplementary figures and images for: Hepatocyte-derived exosomal miR-146a-5p inhibits hepatic stellate cell EMT process: a crosstalk between hepatocytes and hepatic stellate cells
Source: Cell Death Discov. 2023 Aug 19;9:304. doi: 10.1038/s41420-023-01602-y (PMC10439924; doi:10.1038/s41420-023-01602-y)

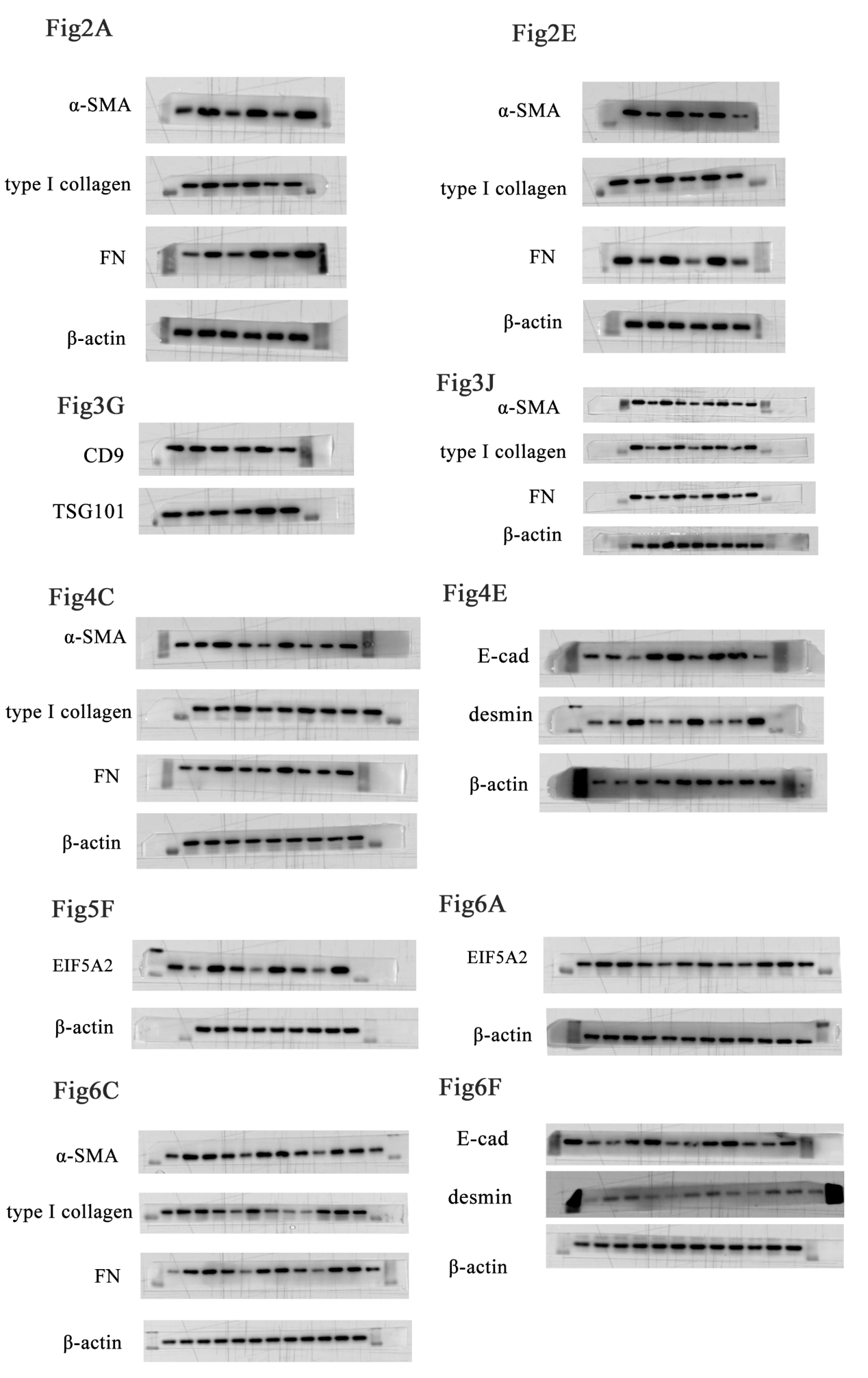

Supplement: Supplementary file 1 — Supplemental Material [file 41420_2023_1602_MOESM1_ESM.docx]
