# Supplementary material for: Hepatocyte-derived exosomal miR-146a-5p inhibits hepatic stellate cell EMT process: a crosstalk between hepatocytes and hepatic stellate cells
Source: Cell Death Discov. 2023 Aug 19;9:304. doi: 10.1038/s41420-023-01602-y (PMC10439924; doi:10.1038/s41420-023-01602-y)
Supplement: Supplementary file 2 — Table S1 [file 41420_2023_1602_MOESM2_ESM.docx]

**Table S1 Primer sequences**

| Gene | Forward sequence | Reverse sequence |
| --- | --- | --- |
| α-SMA  (Mouse) | 5'-GCCATCTTTCATTGGGATGGA-3' | 5'-CCCCTGACAGGACGTTGTTA-3' |
| Col1A1  (Mouse) | 5'-CGATGGATTCCCGTTCGAGT-3' | 5'-GAGGCCTCGGTGGACATTAG-3' |
| FN  (Mouse) | 5'-CTTTGGTGCAGCACAACTTC-3' | 5'-CCTCCTCGAGTCTGAACCAA-3' |
| EIF5A2  (Mouse) | 5'-GAAACTGGCGAAGTCCGTGA-3′ | 5'-CACATGACAGACACCTGCACA-3′ |
| β-actin (Mouse) | 5'-GTGCTATGTTGCTCTAGACTTCG-3' | 5'-ATGCCACAGGATTCCATACC-3' |
| miR-146a-5p  (Mouse) | 5'-CGAGTCCAGTTTTCCCAGGA-3' | 5'-GTCGTATCCAGTGCAGGG-3' |
| miR-1191a  (Mouse) | 5'-CGCGCGCAGTCTTACTATGTA-3' | 5'-ATCCAGTGCAGGGTCCGAGG-3' |
| U6  (Mouse) | 5'-GAAGATTTAGCATGGCCCCTGC-3' | 5'-CAGTGCAGGGTCCGAGGT-3' |
| EIF5A2  (Human) | 5'-AAGATGGTTACCTTTCCCTG-3' | 5'-TACAGCATATTCTTCACTCATTG-3' |
| miR-146a-5p  (Human) | 5'-CGCGTGAGAACTGAATTCCA-3' | 5'-ATCCAGTGCAGGGTCCGAGG-3' |
| β-actin  (Human) | 5'-CCTGGCACCCAGCACAAT-3' | 5'-GGGCCGGACTCGTCATAC-3' |
| U6  (Human) | 5'-AGAGAAGATTAGCATGGCCCCTG-3' | 5'-CAGTGCAGGGTCCGAGGT-3' |
